# Biochemical Predictors of Response to Neoadjuvant Therapy in Pancreatic Ductal Adenocarcinoma

**DOI:** 10.3389/fonc.2020.00620

**Published:** 2020-05-12

**Authors:** Seifeldin Awad, Ahmad M. Alkashash, Magi Amin, Samantha J. Baker, J. Bart Rose

**Affiliations:** ^1^Department of Surgical Oncology, University of Alabama in Birmingham, Birmingham, AL, United States; ^2^Department of Gastroenterology, Cairo Fatimid Hospital, Cairo, Egypt

**Keywords:** pancreatic duct adenocarcinoma (PDAC), neoadjuvant chemotheraphy, CA 19-9–carbohydrate antigen 19-9, biomarker (development), tumor biomarker

## Abstract

Pancreatic ductal adenocarcinoma (PDAC) is becoming increasingly more common. Treatment for PDAC is dependent not only on stage at diagnosis, but complex anatomical relationships. Recently, the therapeutic approach to this disease has shifted from upfront surgery for technically resectable lesions to a neoadjuvant therapy first approach. Selecting an appropriate regimen and determining treatment response is crucial for optimal oncologic outcome, especially since radiographic imaging has proven unreliable in this setting. Tumor biomarkers have the potential to play a key role in treatment planning, treatment monitoring, and surveillance as an adjunct laboratory test. In this review, we will discuss common chemotherapeutic options, mechanisms of resistance, and potential biomarkers for PDAC. The aim of this paper is to present currently available biomarkers for PDAC and to discuss how these markers may be affected by neoadjuvant chemotherapy treatment. Understanding current chemotherapy regiments and mechanism of resistance can help us understand which markers may be most affected and why; therefore, determining to what ability we can use them as a marker for treatment progression, prognosis, or potential relapse.

## Introduction

Pancreatic ductal adenocarcinoma is the 4th leading cause of cancer death in the United States and has recently been associated with an increased incidence (1). The majority of patients with PDAC present with unresectable disease, whether locally advanced or metastatic. Thus, new modalities in early detection and treatment response are necessary. Most large centers are now treating patients with borderline resectable or locally advanced disease with upfront (neoadjuvant) chemotherapy and resecting if no progression occurs. However, the desmoplastic and fibrous nature of PDAC can make the interpretation of surveillance imaging problematic when trying to determine treatment response (2). For instance, in patients with PDAC who underwent neoadjuvant FOLFIRINOX therapy, cross-sectional imaging was not reliable tool to predict resectability or pathologic treatment response (3). Therefore, a combination of tumor biomarkers and traditional imaging methods maybe a better tool for determining treatment response and resectability.

Molecular profiling represents a promising avenue of personalized cancer care that may be prognostic of oncologic outcomes. Using these techniques in a thoughtful way can guide us to creating individualized treatment plans for each patient based on their tumors molecular profile. This concept was recently tested in a clinical trial assessing the utility of molecular profiling as a guide for neoadjuvant regimen choice in patients with PDAC. Using a panel of 6 protein biomarkers to guide gemcitabine or 5-FU based chemotherapeutic selection, the authors increased rates of treatment completion (neoadjuvant and resection) from 50 to 70% in borderline resectable PDAC cases, and from 80 to 90% in resectable cases. These improvements translated to overall survivals (OS) of 38 months in all patients and 45 months in patients who completed treatment, both an improvement over historical controls (4).

Biomarkers can be any combination of biochemical, radiographic, or pathophysiologic variables utilized to provide clinically useful diagnostic or prognostic information. Ideally, biomarkers should be relatively easy to obtain (no biopsy needed), inexpensive, reliable, and provide actionable information (5). This article will review current neoadjuvant chemotherapeutic approaches in PDAC and a variety of promising diagnostic and prognostic biochemical biomarkers in this patient population.

## Common Neoadjuvant Drugs Regimens

### FOLFIRINOX

FOLFIRINOX is a combination of 5-fluorouracil (5FU), leucovorin, irinotecan and oxaliplatin. 5FU targets thymidelate synthase (TS), which converts deoxyuracil monophosphate (dUMP) to deoxythymidine monophosphate (dTMP) and requires N5 N10-methylene tetrahydrofolate as a cofactor. Inhibition of TS results in a decreased pool of the nucleoside thymidine, which is toxic to rapidly dividing cells. The 5-FU-TS complex is stabilized by the reduced folic acid leucovorin. 5-FU is catabolized by dihydropyrimidine dehydrogenase (DPD). Irinotecan is a pro-drug for the topisomerase-1 inhibiting molecule SN-38. Oxaliplatin works by preventing DNA replication and transcription through formation of DNA crosslinks (6) leading to cell death.

### Gemcitabine and Nab-Paclitaxel

Gemcitabine (GEM) has several mechanisms of action, and therefore several potential targets for resistance; GEM acts as a pyrimidine nucleoside and it is activated intracellularly by deoxycytidine kinase (dCK). PDAC can down regulate dCK via increased CYR61/CCN1 signaling, resulting in GEM resistance (7). GEM enters the cell via a facilitated transport mechanism involving the trans-membranous protein human equilibrative nucleoside transporters (hENT1). Patients who are negative for hENT1 have worse OS and prognosis than hENT1 positive patients when given GEM based chemotherapy (8). It has been shown that GEM effectiveness can be altered by changes in expression of the translation inhibiting microRNA miR663a (9).

Taxols are chemotherapeutic drugs that bind to α-tubulins and hyperstabilize microtubules, thereby impairing cell division. Neoadjuvant therapy with GEM and docetaxel has been associated with high resection rates, favorable surgical outcomes, and potentially improved survival in patients with PDAC (10). When paclitaxel is conjugated to albumin (NP), it has increased solubility, improved cellular uptake, and decreased risk of hypersensitivity reactions. Clinical trial data in the metastatic setting has shown GEM-NP in combination is superior to GEM alone (11).

### Cisplatin and Platinum Based Therapies

Cisplatin and platinum-based therapies are widely used in the treatment of PDAC and have been studied as a single treatment or in combination with other chemotherapy drugs (such as gemcitabine) (12). Cisplatin works to induce apoptosis in the cancer cells and when combined with other drugs can further increase DNA damage (13–15). In PDAC, cisplatin has shown limited improvement when combined with other therapies secondary to resistance. Cancer cells can become resistant to cisplatin through several mechanisms including drug transport, DNA damage response, DNA repair, and modulation of apoptosis (16, 17).

## Biomarkers with current Clinical Use

### Carbohydrate Antigen 19-9 (CA 19-9)

This molecule is a Sialylated Lewis (a) cell surface antigen that plays an important role in mediating leukocyte migration along endothelial surfaces by binding to P-selectin. This particular mechanism of action has also been proposed to play a role in PDAC metastasis through extravasation of tumor cells (18). CA 19-9 has been recognized as a potential diagnostic biomarker by the National Comprehensive Cancer Network (NCCN). These guidelines recommend that levels should be measured before each stage of treatment: neoadjuvant therapy, surgery, adjuvant therapy, and then every 3–6 months for up to 2 years post-treatment for follow up (19). However, there are still several limitations associated with this biomarker that will be discussed below. Initially elevated levels above 1,000 U/mL have been shown to have poorer survival in upfront resectable disease, but this has not translated to the neoadjuvcant setting. In fact, recent work has found that any elevation in CA19-9 in early stage disease, has a worse survival than patient with normal levels, and suggested these patients be considered for neoadjuvant treatment (20, 21).

In the neoadjuvant setting, normalization of CA 19-9 after upfront chemotherapy was associated with longer OS in patients who did not undergo tumor resection (15 vs. 11 months; *p* = 0.022). Similarly, normalization of CA 19-9 level in patients who underwent subsequent resection was also associated with longer OS (38 vs. 26 months; *p* = 0.020) (22) CA 19-9 may also be prognostic of resectability in patients with PDAC. It has been reported that patients with a median CA 19-9 level of <130 U/mL are more likely to have resectable tumors than patients with higher levels (23).

If ordered before each stage of treatment as the NCCN recommends, CA 19-9 levels can be followed throughout treatment and give information about prognosis. Pretreatment CA 19-9 level >1,200 U/mL, a post-treatment CA 19-9 >100 U/mL, and a CA 19-9 level that declines <40% following chemo-radiotherapy may possibly serve as a surrogate marker for poor survival in advanced PDAC (24). The group with the higher pre-treatment level had a mean survival time of 8 months compared to 13 months for the group with pretreatment levels of <1,200 U/mL. After GEM, a CA 19-9 level that drops that >20% after 8 weeks is associated with improved OS (383 days) compared to patients who had a rise in their CA19-9 level or a decline of <20% (OS 281 days) (25). Patients with pathological complete response had a post gemcitabine, docetaxel, and capecitabine therapy CA19-9 level of 18.5 U/ml (26).

Finally, CA 19-9 can be used in the post resection surveillance period for detecting early disease recurrence. CA 19-9 levels higher than 125 and 200 U/mL have both been shown to be indicative of tumor recurrence following pancreatic resection (27, 28).

Of note, CA19-9 may be falsely elevated with biliary obstruction, biliary endoprotheses, and/or cirrhosis. Another limitation is the fact that up to 10% of population does not express the CA19-9 antigen due to lack of the fucosyltransferase enzyme (29).

### Carcinoembryonic Antigen (CEA)

CEA is a surface glycoprotein that facilitates cell adhesion. It is normally produced by the fetal gastrointestinal tract and its production is halted before birth with postnatal levels declining to below 20 ng/ml in adults. CEA levels can be elevated in colorectal adenocarcinoma and other adenocarcinomas including PDAC. CEA is equally specific (85 vs. 83%), but less sensitive (44 vs. 73%) than CA 19-9 for PDAC (30), making it less useful diagnostically. In patients with known PDAC, elevated CEA (>4.45 ng/mL) was associated with early recurrence (27). CEA may be most helpful when patients present with other benign pancreatic conditions, discordant radiological findings, and elevated CA 19-9 levels (30). CEA is frequently elevated in smokers (31), patients with hypothyroidism, and other malignancies. Liver failure can also compromise CEA catabolism and lead to falsely elevated levels.

## Proposed Biomarkers With Limited Clinical Use

### Cancer Antigen 125 (CA-125 or MUC16)

CA-125 is a mucin glycoprotein expressed at cell surfaces and plays a role in the barrier immunity of mucosal surfaces. Due to its abundant presence at various mucosal surfaces, this marker lacks specificity. In practice, its role is mainly limited to the treatment algorithm of ovarian cancer. CA-125 binds to mesothelial cell surface proteins and may have a role in ovarian cancer metastasis to the peritoneum. This marker is of interest because this mechanism of action may help researchers gain insight into how PDAC tumor cells form peritoneal metastases (32).

While CA 19-9 is more sensitive in the general population, CA-125 may have a superior specificity for detecting PDAC in diabetics (33). Studies have suggested using CA-125 in a panel with CA19-9, CEA, and CA242 improves diagnostic accuracy from 68% alone to 92% in combination (34). However, this data has not been validated in a prospective manner. CA−125 can be elevated in the setting of biliary obstruction, which is common in patients presenting with PDAC.

### Cytokeratin 19-Fragments (CYFRA 21-1)

Cytokeratin is a key component of the cellular cytoskeleton and plays a role in cell stability. CYFRA 21-1 is a fragment of cytokeratin 19 and is measurable in the serum of patients with PDAC. CYFRA 21-1 levels have been correlated with OS, burden of disease, and response to treatment (35).

### S-Pancreas-1 Antigen (SPAN1)

SPAN1 is a glycoprotein expressed in PDAC with limited clinical use. The sensitivity of combination assays of Span-1 were 84% with CA19-9 and 86% with CEA, (36). Other authors suggest that SPAN1 may be useful in early detection of treatment failure of GEM therapy in patients with PDAC (37). SPAN1 levels higher than 37 U/mL may be an independent risk factor for early recurrence and LN metastases (28). SPAN1 is falsely positive with cirrhosis and hepatitis.

### Fecal Elastase-1 (FE1)

Elastase is a serine protease secreted in pancreatic juice and often utilized as an indicator of pancreatic exocrine function. As PDAC is commonly associated with fibrosis, the exocrine function of pancreas can be affected even without evidence of pancreatic duct obstruction. FE1 levels may provide insight to the degree of fibrosis that exists in patients with PDAC (37). Low FE1 serum levels (<200 mg/g) have been associated with 1- and 3-year DFS (disease free survival) rates of 66.2 and 36.6%, while high serum levels(≥200 mg/g) had DFS of 29.9 and 8.8%, respectively (*p* < 0.001) (37).

### Human Equilibrative Nucleoside Transporter 1 (hENT1)

Human equilibrative nucleoside transporter 1 is a membrane associated protein that aids in the uptake of nucleosides and facilitates the intracellular transport of GEM. There is marked variation of hENT1 expression throughout the general population with some individuals expressing negligible levels. This variation in expression can be associated with GEM treatment resistance. A meta-analysis of 10 studies and found that high hENT1 expression was associated with longer OS (HR = 0.37; 95%CI 0.28–0.47) and DFS (HR = 0.44; 95%CI 0.33–0.59) in patients receiving GEM-based adjuvant therapy (38).

### Deoxycytidine Kinase (dCK) and CYR61/CCN1

The protein dCK mediates the active form of GEM via phosphorylation. The CYR61/CCN1 signaling pathway down regulates dCK and up regulates CTGF (connective tissue growth factor). This promotes fibrosis and increases GEM treatment resistance by reducing its phosphorylation. In addition, epithelial-mesenchymal transition (EMT) is induced by CYR61/CCN1, which causes epithelial cells to convert to mesenchymal stem cells. In this transition the cells loose E-cadherin and cell-to-cell adhesion, allowing for invasion through basement membranes, and subsequent metastasis (7). Given this, high dCK expression has been associated with longer OS with GEM-based adjuvant treatment (HR = 0.40; 95%CI 0.20–0.80) and DFS (HR = 0.41; 95%CI 0.22–0.74) (38).

### Ribonucleotide Reductase (RRM1/2)

In addition to GEM's role in DNA polymerase inhibition, GEM also inhibits riboneucluoreductase, an enzyme required for DNA synthesis. Ribonucleotide reductase consists of two subunits, M1 (RRM1 gene) and M2 (RRM2 gene). Overexpression of RRM genes increase concentration of deoxynucleotide triphosphate (dNTP), which out compete GEM for incorporation into DNA, thereby decreasing its effectiveness (39). Additionally, over expression of RRM1 (the regulatory subunit of ribonucleotide reductase) may bind to GEM irreversibly leading to its deactivation (40, 41). In a study which investigates over 40 patients with PDAC, the median OS of RRM1-negative and RRM1-positive patients was 12.9 and 5.1 months, respectively (*P* < 0.05) (42).

### Cytidine Deaminase (CDA) and Deoxycytidylate Deaminase (DCD)

CDA and DCD are enzymes involved in the pyrimidine salvage pathway. High expression of CDA will accelerate GEM catabolism and lead to treatment resistance (43, 44). This makes detection of CDA & DCD in the pretreatment setting beneficial and can aid in predicting potential survival benefit and toxicity before GEM administration (45). In theory, patients with PDAC who have elevated levels of CDA undergoing GEM treatment, may be given Nab-paclitaxel concurrently to induce CDA damage by reactive oxygen species and enhance treatment effectiveness. The most predictive CDA level needs further clinical correlation.

### Dihydropyrimidine Dehydrogenase (DPD)

Dihydropyrimidine dehydrogenase is the rate limiting enzyme in pyrimidine catabolism, whereby thymidine and uracil are reduced. This pathway is a similar to the pathway of 5FU catabolism. DPD activity has been demonstrated to be low in patients with well-differentiated types of PDAC when compared to patients with poorly differentiated and anaplastic types (46). In that same study of 18 patients, low DPD activity (<395 pmol/mg protein) was associated with a significantly improved OS (46). Combining a DPD inhibitor to fluoropyrimidine regimens has been shown to increase the treatment response rate in gastric cancer (47). The DPD inhibitor gimeracil has been studied in pancreatic cancer as a component of the combination treatment S-1 (includes 5-FU prodrug tegafur and potassium oxonate), with the JASPAC-01 clinical trial in Japan showing non-inferiority over single agent GEM in the adjuvant setting with improved 5-yr OS (44 vs. 24%) (48).

### Thymidelate Synthase (TS)

High TS levels may also contribute to gemcitabine resistance; as TS provides an alternative source of substrate for DNA synthesis. This is supported by studies demonstrating a decrease in GEM resistance when TS enzyme expression is knocked down using small interfering RNA (49).

### Multidrug Resistance-Associated Proteins (MRPs)

Multidrug resistance-associated proteins are a member of the ATP-binding cassette (ABC) transporter family and actively transport molecules across the cell membrane (50). Overexpression of MRP4 confers resistance to 6-mercaptopurine (6-MP) and 6-thioguanine (6-TG). Whereas, overexpression of MRP5 confers significant resistance to 5FU (51).

### MicroRNA (miRNA)

miRNA are small non-coding RNA that bind to and inhibit the translation of mRNA. miRNA can act as either a tumor suppressor or oncogene depending on the mRNA it is inhibiting and are variably expressed in PDAC (52) miR211 down regulates RRM2 expression, increasing sensitivity to GEM (53). On the other hand, miR-17-5p inhibition enhances sensitivity to gemcitabine via up regulation of Bim (apoptotic promoter) expression in pancreatic cancer cells (54).

### Excision Repair Cross-Complementing Gene-1 (ERCC1)

ERCC1 acts as a part of nucleotide excision repair pathway. High ERCC1 expression in patients with PDAC has been associated with reduced recurrence free survival (6 vs. 10 months; *P* = 0.03) and decreased OS (9 vs. 18 months; *P* = 0.019) (53, 55).

### Cell-Free DNA (cfDNA)

PDACs can shed DNA fragments into the circulation from necrotic and apoptotic cells in the form of cfDNA. cfDNA has been shown in some studies to predict early tumor recurrence compared to cross sectional imaging (56). It may also be beneficial to monitor treatment response in the metastatic setting (56). In addition to detecting cfDNA in circulation, these fragments of DNA can be tested for mutations known to be associated with PDAC. KRAS mutations detected in cfDNA from patients with PDAC were associated with a 90% chance of disease progression within a median follow-up time of 3.7 months compared to only 25% in the KRAS negative group (*P* = 0.01) (57). Additionally, cfDNA may assist in detecting changes in tumor genetics longitudinally, in that 78% of mutations found in cfDNA after metastatic progression were not detected in the primary tumor at time of resection (58). This suggests that cfDNA may provide a more accurate genomic representation of disease compared to a conventional single site biopsy.

### Circulating Tumor Cells (CTC)

CTC are cells released into circulation from the tumor. Patients who have detectable CTC tend to have a worse prognosis. Detection of epithelial CTCs was significantly associated with worse survival compared with patients without CTCs (median survival 13.7 mo vs. >14 month, *P* = 0.008) (59). CTCs may also provide information on response to neoadjuvant treatment and be prognostic of recurrence post resection up to 2 months earlier than imaging evidence (60).

### Mothers Against Decapentaplegic Homolog 4 (SMAD4)

SMAD4 is a signal transducer for the transforming growth factor beta pathway, which in turn plays a role in cellular proliferation, differentiation and apoptosis. Detection of high levels of SMAD4 is highly correlated with a poorer prognosis (61). Thus, determining SMAD4 status at initial diagnosis may be of value in stratifying patients into local vs. systemic treatment regimens (62).

### Carboxylesterase 2 (CES2)

CES2 is a carboxylase enzyme capable of converting irinotecan into its active form of SN-38. Higher expression of CES2 was associated with increased OS in patients who underwent neoadjuvant FOLFIRINOX treatment (hazard ratio = 0.14, 95% confidence interval = 0.04–0.51, *P* = 0.02) (63).

### Secreted Protein Acidic and Rich in Cysteine (SPARC)

SPARC is a glycoprotein that is overexpressed in the stromal cells of PDAC. SPARC plays a role in cellular proliferation and angiogenesis through the regulation of macromolecular movement, and therefore may assist in determining a tumor's invasive capability (64). SPARC also facilitates the uptake of albumin bound paclitaxel (NP). This may help drug delivery to the hypoxic PDAC microenvironment (65). Accordingly, high SPARC expression is associated with lower survival (11.5 months in case of high expression vs. 25.3 months in case of low expression; *p* = 0.020) (66).

### Epidermal Growth Factor Receptor mEGFR (EGFR)

EGFR (ErbB1, HER1) is a transmembrane tyrosine kinase receptor with downstream targets affecting both DNA replication and cell proliferation. An increased expression of these receptor in the tumor microenvironment will result in increased replication and has been associated with worse clinical outcomes. Patients with resected tumors that stained positive for EGFR by immunohistochemistry had worse OS compared to patients who were negative (18 vs. 34 months, *P* = 0.04) (67).

Erlotinib targets EGFRs and has demonstrated mixed clinical promise in PDAC patients with EGFR positive tumors. In one randomized trial of patients with advanced PDAC receiving GEM therapy alone or GEM plus erlotinib, single agent GEM had worse OS (5.9 vs. 6.2 months) (68). However, another randomized trial comparing GEM vs. GEM plus erlotinib given in locally advanced pancreas cancer, followed by either continuation of chemotherapy or switching to 54Gy radiation with capecitabine found no difference in an intention to treat anlaysis of OS between the GEM and GEM plus erlonitib (13.6 vs. 11.9 months) (69).

### BRCA Mutations

Patients with germline mutations in BRCA are at an increased risk for PDAC (70). Recent phase I clinical trials have shown antitumor effectiveness with platinum-based combination therapy (specifically cisplatin in combination with GEM and veliparib) in patients with PDAC and BRCA mutations. Randomized phase II trails are currently enrolling to study the effect of cisplatin and GEM with and without veliparib for patients with PDAC and BRCA mutations (71, 72).

## Conclusion

Biomarkers may be used to offer patients personalized care and improve oncologic outcomes. While CA 19-9 is currently the only PDAC related biomarker with proven clinical utility, other biomarkers discussed in this review show significant promise to improve care. Further research and breakthroughs in the field of precision medicine will allow us to gain more insight into the role that these and other biomarkers will play in future cancer care.

## Author Contributions

SA, AA, and JR were responsible for data gathering, data review, mansucript drafting, and editing. SB and MA were responsible for editing, reviewing, and formatting the manuscript.

## Conflict of Interest

The authors declare that the research was conducted in the absence of any commercial or financial relationships that could be construed as a potential conflict of interest.
